# The Effects of Prenatal Stocking Densities on the Fear Responses and Sociality of Goat (*Capra hircus*) Kids

**DOI:** 10.1371/journal.pone.0094253

**Published:** 2014-04-07

**Authors:** Rachel M. Chojnacki, Judit Vas, Inger Lise Andersen

**Affiliations:** Department of Animal and Aquacultural Sciences, Norwegian University of Life Sciences, Ås, Norway; Queen Mary, University of London, United Kingdom

## Abstract

Prenatal stress (stress experienced by a pregnant mother) and its effects on offspring have been comprehensively studied but relatively little research has been done on how prenatal social stress affects farm animals such as goats. Here, we use the operational description of ‘stress’ as “physical or perceived threats to homeostasis.” The aim of this study was to investigate the prenatal effects of different herd densities on the fear responses and sociality of goat kids. Pregnant Norwegian dairy goats were exposed to high, medium or low prenatal animal density treatments throughout gestation (1.0, 2.0 or 3.0 m^2^ per animal, respectively). One kid per litter was subjected to two behavioral tests at 5 weeks of age. The ‘social test’ was applied to assess the fear responses, sociality and social recognition skills when presented with a familiar and unfamiliar kid and the ‘separation test’ assessed the behavioral coping skills when isolated. The results indicate goat kids from the highest prenatal density of 1.0 m^2^ were more fearful than the kids from the lower prenatal densities (i.e. made more escape attempts (separation test: P < 0.001) and vocalizations (social test: P < 0.001; separation test: P < 0.001). This effect was more pronounced in females than males in the high density (vocalizations; social test: P < 0.001; separation test: P  =  0.001) and females were generally more social than males. However, goat kids did not differentiate between a familiar and an unfamiliar kid at 5 weeks of age and sociality was not affected by the prenatal density treatment. We conclude that high animal densities during pregnancy in goats produce offspring that have a higher level of fear, particularly in females. Behavioral changes in offspring that occur as an effect of prenatal stress are of high importance as many of the females are recruited to the breeding stock of dairy goats.

## Introduction

It is well known and accepted that stress experienced by pregnant mothers (both human and non-human) can have detrimental effects on embryonic survival and development. Avishai-Eliner et al. [1] define ‘stress’ as “physical or perceived threats to homeostasis“ and Braastad [2] further defines ‘prenatal stress’ as “stress experienced by the pregnant mother which affects the development of the offspring.” Prenatal stress can induce chronic physiological responses similar to defensive responses induced by perceived danger [3]. Exposing a fetus to an increase in stress hormones due to maternal stress affects numerous aspects of fetal brain development and functioning as well as physiology, immunology and behavior [4]–[6]. Multiple studies have shown the negative effects of prenatal stress on the behavioral and cognitive development of the offspring (for reviews see [2], [7]–[9]). Effects such as increased anxiety/emotional reactivity [10], [11] and non-directed locomotive behavior [12] as well as impaired immune system [13], development [14], learning ability [15] and sexual behavior [15]–[18] can be permanent and the effects can survive across generations [19]. This is of great importance for the quality of the breeding stock.

Most farm animal species, including goats, are highly gregarious and social relationships are very important for group cohesion [20]–[23]. However, the relationship animals have with their conspecifics can also be one of the largest sources of stress [5], [23], [24]. Events of aggression between animals can increase as resources, such as space, are diminished [23], [25]. Farm animals often experience prenatal social stress due to routine farm management practices such as disturbances in the dominance hierarchy/social stability and group size and high animal densities [2]. Andersen et al. [11], [26], [27], Barroso et al. [28], Patt et al. [29] and Vas et al. [30] have demonstrated that goats are sensitive to aspects of their social environments such as group size, social stability and space allocation. Yet, due to the lack of studies coupled with the lack of regulations on space requirements for goats in many countries in Europe [30], goats are often kept in densities higher than 1.0 m^2^. Furthermore, studies of prenatal stress most commonly use non-social stressors such as restraint [6], [15], [31]–[36], exposure to bright lights [16], [32], [37], [38] or loud noises [12], [39] or injections of various substances [4], [39]–[43], among others. As Kaiser and Sachser [7] point out, however, the manner in which most stressors are applied in the experimental setting do not exist in the animal's natural habitat. Therefore, social stress may be one of the most biologically relevant stressors [7]. Routine farming practices and the recent intensification of animal production have put great strains on farm animals [28] particularly by disrupting social relationships. Yet relatively little research has been done on how prenatal social stress effects farm animals such as goats.

The focus of prenatal stress and its effects on offspring has been on rodents and non-human primates [2], [8]. However, results from one species can not necessarily be extrapolated to another. Many aspects of development, both pre- and post- natally, can differ drastically between species [44]. For example, although rats (R*attus*) and primates are altricial, several major brain developmental events which happen during the final stages of gestation in rats occur during the first half of gestation in primates [8]. Ruminants are more precocious than both rats and primates and many more neurological and neuroendocrine developmental events occur prenatally [42], [45], [46]. For example, the sensory functions (such as olfactory, which is essential for the development of the mother-young bond [47], sexual behavior [21] and identifying group members [48]) as well as the hypothalamic–pituitary–adrenocortical axis (HPA) (which can be damaged as a result of stress inflicted on the mother (see review by Weinstock [49]), are almost fully developed at birth in lambs and kids [42]. Additional to developmental differences, ruminants have a different placental structure than rodents with the placenta acting as a stronger barrier to maternal hormones [42]. Therefore, the stress responses directly experienced by the mother may not be transferred to the fetus via the placenta in ruminants. Finally, although goats are often put in the same category (behaviorally and physiologically) as sheep, there are differences even among these ruminants. As an example, the young of these two species use different anti-predator strategies. Like the majority of ungulates [50], goats are often categorized as a “hider” species as soon after parturition newborn kids move away from their mothers hiding themselves from potential predators [51]. In contrast, lambs are mobile and follow their mothers shortly after birth (“follower” species) [52] allowing them to flee from predators. Therefore, even post-natally, kids and lambs may experience the same stressors differently and behaviorally react in different ways.

Behavioral reactivity to social stress can be associated with coping ability in chronically stressful environments. How well an animal can cope with stress in its environment can directly affect an animal's ability to survive and reproduce [2], [7], [25]. Evolutionary biologists interpret prenatal stress as the influence the mother has on the developing fetus [7]. In evolutionary terms, the mother should aim to produce offspring that are adapted to the present environment. Should the mother be stressed throughout her gestation then it is optimal for her offspring to be less sensitive to non-fatal stressors [1], [2], [7], [11]. A previous study using social instability as a source of prenatal stress in dairy goats, showed that kids from unstable groups were more fearful (i.e. more escape attempts) in the first exposure to a social test and tended to be more active in seeking contact with a novel object and unfamiliar stimuli kids than those born from goats that were kept in stable groups during pregnancy [11]. A more excitable behavioral style in the offspring was also documented during high population densities in wild populations of guinea pigs (*Cavia aperea*) [53] and similar results have been found in goat kids [42], lambs (*Ovis aries*) [54], [55] and blue foxes (*Alopex lagopus*) [56] that were prenatally subjected to other types of stressors such as isolation, transport and human handling. Goat kids begin to shift from their siblings as a preferred companion [57] to develop relationships with other similar aged conspecifics by 7 days of age [11], [51]. In addition, they show vocal convergence with the similarly aged group mates by 5 weeks of age [58] and go on to spend twice as much time with their siblings or similar aged conspecifics than with their mothers during the first 15 weeks of life [57]. Therefore, if there are either adaptive or maladaptive effects of prenatal stress on the kid's early social skills they are most likely to be manifested early in the kid's life.

The aim of this study was to investigate the prenatal effects of different herd densities in Norwegian dairy goats on fear responses during separation and the sociality of goat kids when presented to social companions in an unfamiliar environment. Results on the mothers indicate that an animal density of 1.0 m^2^ during pregnancy resulted in more social stress in terms of an increased number of agonistic interactions than animals in densities of 2.0 m^2^ or 3.0 m^2^. Based on previous findings indicating a more excitable behavioral style in offspring subjected to prenatal stress, we predicted that in a situation where they are separated from their mother and group mates goat kids from a high prenatal density would show more active fear responses such as a higher number of escape attempts and vocalizations. These are well documented signs of fear and stress during isolation tests in many species including goats [11], [42], sheep [54], [59] and cattle (*Bos taurus*) [22], [60]. In addition, studies have shown prenatal stress results in juveniles exhibiting less normal social behaviors in rats [61], [62] and monkeys [63], [64]. Therefore, we predicted kids from a high prenatal density would have a weaker motivation to seek social contact with companion kids, especially with a familiar one (i.e. longer latencies to seek contact, fewer contacts made and less time spent in close proximity to companions) and be less capable of distinguishing between a familiar and non-familiar stimulus kid than kids born from goats kept at lower animal densities during pregnancy.

## Materials and Methods

### Experimental design

This work was part of a large EU project called the Animal Welfare Indicators project (AWIN; http://www.animal-welfare-indicators.net). Results on the goat mothers, reported in Vas et al. [46], showed goats kept at higher densities had an increase in agonistic behaviors but the treatments did not have an effect on socio-positive behaviors (behaviors which facilitate cooperation and group cohesion). Results on the cognitive abilities of the goat kids indicate that they are able to perform advanced stages of Piagetian object permanence tasks (Chojnacki et al., manuscript in preparation).

### Ethics Statement

Animals were recruited from the experimental goat herd of the Norwegian University of Life Sciences, Ås, Norway. The herd is managed in a way that is typical of commercial Norwegian dairy goat farms. Ethical rules stated by Forsøksdyrutvalget (the Norwegian committee for research animals (FDU), www.fdu.no) which satisfy the European Union (EU) animal testing directive (86/609/EEC), the Council of Europe Convention on laboratory animals (ETS 123; http://conventions.coe.int/Treaty/en/Treaties/Html/123.htm) and the legislations for keeping farm animals and small ruminants in Norway (www.mattilsynet.no) were followed. In addition, all study practices were reviewed and approved by the Norwegian University of Life Sciences institutional animal care and use committee, the Animal Production Experimental Centre (Senter for husdyrforsøk (SHF)). As the experiment did not expose the goats to conditions other than what is common practice for the keeping of dairy goats in Norway and the EU a specific protocol approval number was not issued.

### Animals and treatment during gestation

Healthy, pregnant, dehorned Norwegian dairy goats (*Capra hircus*), age 2.8 ± 0.1 years (range 2–5 years) and weighing on average 50.2 ± 1.0 kg (range 36.4–68.5 kg) were used in the experiment. This herd spends the summer periods on pasture in the mountains and they are all familiar with each other. From the time the goats were transported from pasture (mid-September, 2011) they were stalled individually when indoors with fencing which allowed visual, olfactory and limited physical access to each other such that communication was minimally impacted. Beginning in mid-October, 2011, the goats were placed into groups of 15–35 and the hay and concentrate provided was reduced in order to terminate lactation. Because the timing of exposure to prenatal stress may have a great influence on the effect of the development of the fetus [2], [8] and because it is difficult to pinpoint which period of goat gestation may be most sensitive to stressors, we wished to expose the mother goats to the treatment from the confirmation of pregnancy throughout the entire gestation period. Therefore, approximately 2 weeks later, at the start of the experiment (early November), 54 multiparous female goats were selected from the herd of 98 individuals based on their confirmation of pregnancy (by not returning to estrus and/or ultrasound investigation 3 to 7 weeks after mating or insemination) and expected time of parturition. The goats were randomly and evenly distributed in herds of six animals (a total of 18 animals per treatment) in densities of 1.0 m^2^, 2.0 m^2^ or 3.0 m^2^ per animal (low density: pens 276 cm × 650 cm each; medium density: pens 189 cm × 632 cm, 224 cm × 540 cm, 276 cm × 435 cm; high density: pens 189 cm × 317 cm, 224 cm × 270 cm, 224 cm × 270 cm, see Vas et al. [30] for specifics on goat allocations and pen densities chosen). The goats were kept in stable groups and not mixed with new individuals throughout their entire pregnancy until weaning of their kids at 6 weeks of age. Data on the effects of the density treatment on the mothers were presented in another study (see Vas et al. [30]).

The treatment pens were indoors, in one of two insulated, mechanically ventilated rooms in the same building. The room temperature was held at approximately 10°C. All pens had 1.5 m high solid walls made of 15 mm plywood, which prevented physical contact between groups, and flooring consisted of expanded metal flooring with a 60 cm deep area at the rear end of the pen made of solid wood with sawdust bedding. The pens were cleaned in the morning and afternoon after feeding and fresh bedding was added as needed to the solid floor area. Artificial lighting provided a 7:17 h light: dark regime with lights on at 8 am in addition to natural lighting through windows along either side of the building.

The goats had free access to fresh water, grass silage and salt blocks with copper. The front of each pen had six individual feeding places which gave access to a common feeding trough. The feed from the previous feeding was cleaned every morning and afternoon and new silage was supplied every morning and afternoon, in addition, the goats were fed 0.2 kg of concentrate each morning for most of the experimental period. The concentrate was gradually increased to 0.5 kg per goat in the last part of pregnancy (from mid-January until kidding). At this time, the feed was also complemented with hay in the afternoon to stimulate the goats' digestion. Kids were born from the beginning of February to the beginning of March. At the time of expected birth (either by showing signs of parturition or if the expected date of parturition had passed), each goat was isolated from the herd until 24 hours after parturition to allow for maternal care and bonding. After the 24-hour post-parturition period, the goats and their kids were returned to their treatment herd and the treatment conditions remained until the kids were removed for weaning at 6 weeks of age. The feed openings in the pens allowed kids to move freely between their home pen and separate kid areas which had solid wooden floors and free access to hay.

One goat from the medium density treatment aborted 16 days before the expected date of parturition. This goat was removed from the experimental pen for 8 days for observation, medicated and returned to the same experimental pen until the end of the treatment. A stillborn kid was born in the medium density treatment (most likely due to complications at birth) and the mother could not be saved. One goat from the low density treatment gave birth to two live and two stillborn kids (the latter two were immature). A live-born singleton kid from the high density treatment had to be removed for a parallel study and it was not used in the behavioral tests. Finally, one kid in the high density was missed for the behavioral tests. Only data from live born kids are presented.

### Behavioral tests

**Figure 1:**
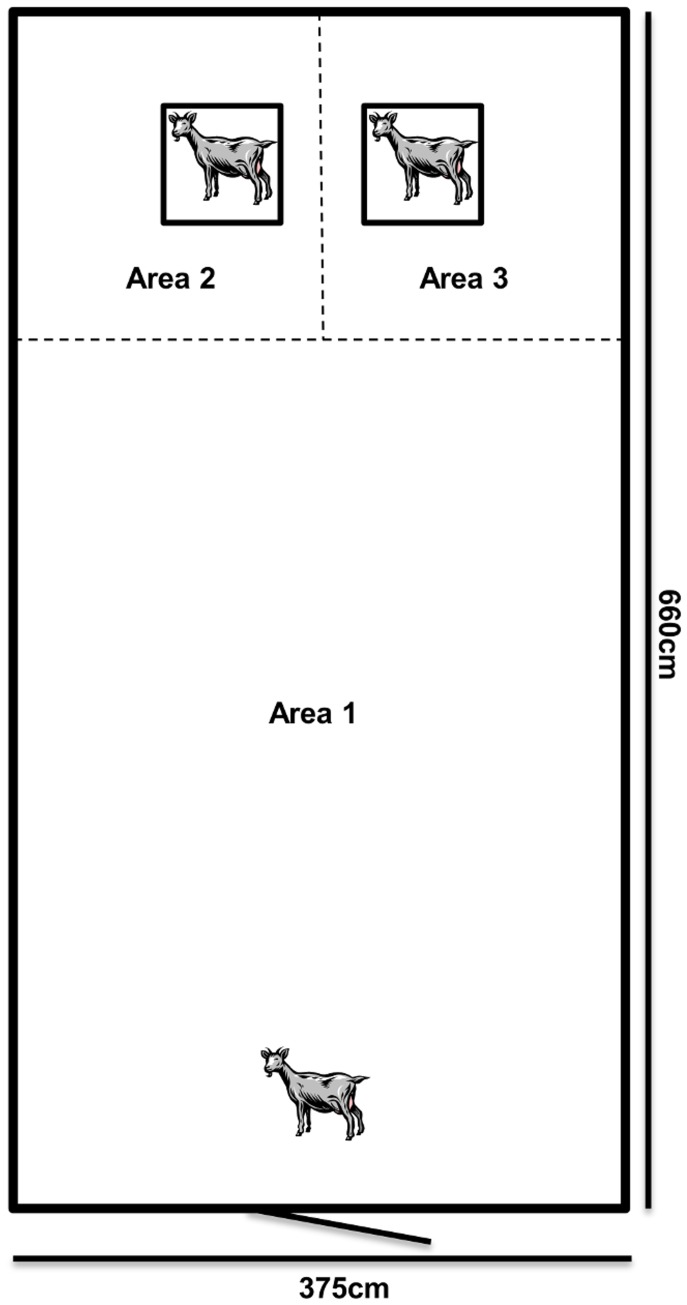
A schematic picture of the test arena. The social test including the two stimuli kids in cages. To test the general sociality of the test kids, areas 2 and 3 were combined. Stimuli kids were removed for the separation test.

One kid from each litter (low density: n  =  18, females  =  9, males  =  9; medium density: n  =  16, females  =  6, males  =  9; high density: n  =  16, females  =  8, males  =  8) was individually subjected to two types of behavioral tests: a ‘social test’ and a ‘separation test’ the week each kid turned 5 weeks of age. The kids were divided into five groups and the testing period was staggered over a 5 week period as there were 5 weeks separating the first birth from the last. Each group tested contained kids from all three treatments. In the case of twin litters, one kid was chosen at random (sex was not controlled for) to avoid any litter effect [65], [66]. Five weeks of age was chosen as disbudding and castration procedures were conducted at 3 weeks of age as per common practice in Norway and this allowed for proper healing from the procedures. The behavioral tests assessed the behavioral responses, preference for familiar versus unfamiliar companion and general sociality of the goat kids when presented with a familiar and unfamiliar kid (social test) and the behavioral coping skills when separated from group mates (separation test). Both behavioral tests were conducted in an unfamiliar test arena (375 cm by 660 cm; Fig. 1) in a separate room but in the same building as the treatment pens. The duration of each behavioral test was 2 minutes. For the social test, 2 minutes was chosen based on previous social recognition studies in farm animals (e.g. goats: [48], [67], sheep: [68], [69], cattle: [70], horses (*Equus caballus*): [71], [72] where social recognition tasks typically lasted 2 minutes. Two minutes was chosen for the separation test as we were interested in the initial behavioral reactions, not behaviors which indicate frustration or acclimatization, in isolation situations. Two portable cameras (SONY HDR-SR12) were set up at either side of the test arena to record behaviors.

### The ‘social test’

The ‘social test’ was a modification of tests used by Boivin and Braastad [67] and Andersen et al. [11] for goat kids. During the test, the kids were subjected to one familiar stimulus kid (not experimental subjects nor a sibling, but of the same kid area from birth with free visual, olfactory, tactile and auditory contact with the test kid) and one unfamiliar stimulus kid (not experimental subjects and of the different kid areas with no previous visual, olfactory, tactile and auditory contact), simultaneously, each placed in a cage (76 cm by 48 cm by 56 cm) with straw bedding located in the test arena (Fig. 1). Similar to the test arenas used in studies of conspecific recognition in sheep [73], [74] and cows [22], the floor in the test arena was divided into three areas: area 1, where the test kid always entered, area 2, where the familiar social stimulus kid was placed and area 3, where the unfamiliar social stimulus kid was placed (Fig. 1). Areas 2 and 3 were alternated for every other test kid to control for side biases. Each stimulus kid was of similar size and coloring; however, we were unable to control for the sex of the stimuli kids. The cages allowed visual, olfactory, auditory and limited tactile contact. Each test kid was gently lifted and carried from its home pen and put into the experimental pen through the entrance door in area 1, which was in the side of the test arena opposite to the stimuli cages. The test lasted 2 minutes from the time the experimenter left and closed the door to the experimental room. An example of a video from the social test is included as a supplementary file (Video S1). The following behavioral variables during this test were measured via video recordings from the two portable cameras:

- Latency (in seconds) to enter the area of each stimulus cage (Fig. 1; areas 2 and 3);

- Time spent (in seconds) in area of each stimulus cage (Fig. 1; areas 2 and 3);

- Which stimulus kid was nose contacted first (familiar or unfamiliar);

- No. of nose contacts made to each stimulus cage;

- No. of escape attempts (when the kid ran towards one of the pen walls and reared/or jumped towards it);

- No. of vocalizations;

The number of vocalizations made by each stimulus kid was counted with the aim of calculating whether the number of vocalizations the stimuli kids made affected which stimulus the test kid contacted first. Finally, to understand the effect of treatment on the general sociality of the kids, we summed the total time spent in the areas of either stimulus (time spent in area 2 plus time spent in area 3) and the total number of contacts made to either stimuli and recorded the shortest latency to enter either stimulus area for each test kid. With the exception of vocalizations and escape attempts, behavioral recordings were missed for the first group tested (n  =  11: low density: n  =  3; medium density: n  =  4; high density: n  =  4) due to one of the cameras malfunctioning.

### The ‘separation test’

Immediately following the social test, two experimenters quietly reentered the test room, gently removed the two stimulus kids from the cages and carried them out of the test room while the test kid remained unrestrained in the test arena. The initial responses during social isolation are commonly used measurements of interpreting how well gregarious animals cope in stressful situation (for example [75]). As for the ‘social test’, the separation test lasted 2 minutes from the time the door closed after the last experimenter left the experimental room. An example of a video from the separation test is included as a supplementary file (Video S2). The following behavioral responses were measured via video recordings from the two portable cameras:

- Total duration (in seconds) of movement;

- No. of escape attempts (when the kid ran towards one of the pen walls and reared or jumped towards it);

- No. of vocalizations

The duration of movement was missed for the first group tested (n  =  11: low density: n  =  3; medium density: n  =  4; high density: n  =  4) due to the camera malfunction.

### Statistical methods

R Statistics version 3.0.2 [76] was employed to run all statistical models.

Data were tested for normality using a Shapiro Wilk test. Most of the variables measured (with the exception of difference in time spent in each stimuli area, difference in the number of contacts made to each stimuli and total number of contacts made to either stimuli in the social test and vocalizations and duration of movement in the separation test) were not normally distributed; therefore, generalized models were used. The random effect of the pen nested within treatment was not significant for any of the variables tested nor was the effect of test group; therefore, the generalized models were simplified to test the effect of fixed parameters. A generalized linear model (GLM) with Poisson distribution and log link was applied to all frequency data (vocalizations, nose contacts and escape attempts), while a GLM with Gamma distribution and identity link was applied to data regarding latency to approach and time spent in each area for the social test and duration of movement in the separation test. All GLM models were calculated as likelihood ratios. For both the social and separation test, treatment and sex were fixed effects. Interactions between treatment and sex were tested and post-hoc Tukey tests were applied to find where the significant effect laid when appropriate.

To investigate the effect of treatment on the social recognition abilities of the goat kids in the social test, we calculated the differences (familiar - unfamiliar) between latencies to enter each stimulus area, time spent in the area of each stimulus and number of contacts made to each stimulus for each test kid and applied a GLM with Gaussian distribution and identity link. Because we were unable to control for the sex of the stimuli kids, the sex of the stimuli kids fell into one of three categories: both stimuli were of the same sex as the test kid, both stimuli were of the same sex but a different sex than test kid or the stimuli were of different sexes. We tested the effect of stimuli sex in all the cases where the two stimuli were not of the same sex (low density: n  =  4; medium density: n  =  3; high density: n  =  5) and, although the numbers are low for proper statistical analyses, we found no effect on treatment (X_2_^2^  =  0.36, P  =  0.55), sex (X_1_^2^  =  1.69, P  =  0.19) or the interaction between treatment and sex (X_1_^2^  =  0.77, P  =  0.38) so this factor was removed from the model.

## Results

**Figure 2:**
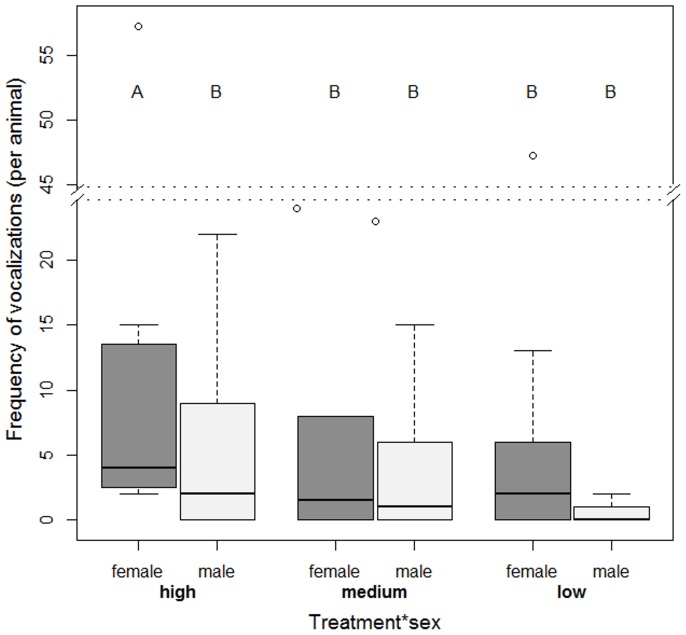
The number of vocalizations made by the goat kids during the social test. The interaction between treatment (high, medium and low densities) and sex (females and males) during the ‘social test’ in the frequency of vocalizations (with median and interquartile range in the box, outliers shown as dots) made by the test kid. Different letters indicate significant differences (P < 0.001).

Significant interactions between treatment and sex were found in both the social and the separation tests. Females from the high density vocalized more than their male counterparts in the high density and both sexes in the other treatments during the social test (Fig. 2). During the separation test, females from the high density also vocalized more than their male counterparts in the high density as well as both sexes in the other treatments, while the females in the medium density vocalized significantly less than the other kids (X_2_^2^  =  39.18, P < 0.001; vocalizations: median (IQR) of females in high: 68.0 (61.5–72.3), medium: 32.5 (18.3–54.25) and low: 60.0 (42.0–63.0) and males in high: 51.0 (40.0–60.0), medium: 55.0 (47.0–57.0) and low: 51.0 (44.0–59.0)). Kids from the high density treatment made significantly more escape attempts (X_2_^2^  =  27.53, P < 0.001) than kids from lower densities in the separation test (escape attempts: median (IQR) of kids in high: 2 (1–4), medium: 0 (0–1) and low: 0 (0–1). Only two individuals (both from the high density) made escape attempts during the social test (with 1 and 8 attempts respectively). Sex did not influence number of vocalizations the test kid made in either test nor how many escape attempts were made in the separation test.

Males were less social than females during the social test as they tended to spend less time in the areas surrounding the stimuli kids (X_1_^2^  =  3.22, P  =  0.07; mean total time spent (seconds) ± SE females: 98.7 ± 4.2 sec; males: 84.7 ± 6.7 sec) and nose contact the stimuli kids fewer times than females (X_1_^2^  =  3.68, P  =  0.05; total number of contact (mean) ± SE females: 9.3 ± 0.7; males: 7.8 ± 0.7). There was a tendency for an effect of treatment in the difference in latency to approach the familiar or unfamiliar kid; however, T-tests revealed kids from the medium density only weakly tended to prefer one of the stimuli kids (the unfamiliar) but kids from high and low treatment densities did not distinguish between the two stimuli (high: t  =  −0.25, P  =  0.81; medium: t  =  −2.0, P  =  0.08; low: t  =  1.0, P  =  0.35). Furthermore, sex and the interaction between treatment and sex were all insignificant in regards to difference in latency to approach the stimuli kids. There was no significant effect of treatment on all other variables measured during the social test. Finally, no variables measured regarding the duration of movement during the separation test were significant.

## Discussion

This study investigated the effects of prenatal social stress via three different herd densities on the fear responses during separation and sociality of goat kids during a social test. As predicted, kids from the high density treatment showed more fear responses (i.e. made more escape attempts in the separation test) than kids from the other treatments. An increase in vocalizations was found in females from the high density treatment during both behavioral tests. Finally, males tended to be less social than females, regardless of treatment, in the social test.

A large amount of data indicates that prenatal stress manifests itself in males and females differently. Females generally appear to display a larger increase in anxiety-like behaviors than males [2], [3], [9], [42], [56] while males show increased learning deficits [3], [9], [32], [39]. In accordance to these studies, males tended to be less social during the social test. While females, particularly from the highest density, showed an increase in vocalizations (a behavior indicative of an anxiety-like state) during both tests. Interestingly, however, females in the medium density vocalized significantly less than all other kids during the separation test. We do not have a logical explanation for this finding. In contrast to the work done by Clarke and Schneider [64] showing prenatal stress caused an increase in the sociality of male rhesus monkeys, but similar to that of Ohkawa [77] on rats, we found an increase in female sociality in goat kids. It may be that female goats are simply more sensitive to separation and social proximity than males. As adults, female goats appear to be more sensitive than males to improvements in their environment [78]. Furthermore, wild female goats remain highly social throughout the year [24], [52], [79] separating for only a brief period while kidding. Once a herd has been formed, it is rare that females migrate into another herd [79]. In contrast, the degree to which males segregate from the herd is highly variable ([80] and reviewed by O′Brien [81]). Lambs begin to show sexual dimorphism in their play behavior [82] and kids begin to synchronize activities such as lying, standing and feeding with adults [57], as early as 5 weeks old. Whether sexual social segregation behavior manifests itself as early as 5 weeks old in male kids should be further investigated.

Goats from the high density treatment produced young that were more likely to make escape attempts. This supports the theory of evolutionary biologists that mothers should produce offspring that are optimally adapted to the present environment [1], [2], [7], [11]. Should the mother be stressed throughout her gestation then it can be optimal for her offspring to be less sensitive to non-fatal stressors that may be a common occurrence in the environment it is born into. Alternatively, if the stressors are life threatening, for example an increased exposure to predators, then it is optimal if the offspring are more sensitive to stressors. In this case, isolation from conspecifics was a key factor in eliciting stronger fear responses as this simulated a situation with an increased threat of exposure to predators. This is further illustrated when the two test situations are compared. Only two kids made escape attempts during the social test, while over half of the kids made escape attempts during the separation test. Furthermore, during the separation test, the average number of vocalization made per kid increased nearly 10-fold when compared to the social test. Porter et al. [59] found similar results in lambs, where the presence of a conspecific, regardless of familiarity, reduced the frequency of bleating. While these results do indicate that separation results in a higher level of fear than when companions are present, it is important to bear in mind that the separation tests were always conducted immediately following the social tests. Therefore, the increase in stress indicators (e.g. vocalizations and escape attempts) may have been a compounded effect. The order of the tests should have been alternated to control for this possibility.

In contrast to what was predicted, the test kids did not seek comfort in their social companions and did not distinguish between the familiar and unfamiliar stimuli kids during the social test. The ability to recognize familiar individuals is crucial in the development and maintenance of social bonds [83]. Multiple studies investigating social recognition tasks have used the differences in responses to the stimuli presented, for example, length of time spent investigating the familiar minus the unfamiliar stimulus as evidence that the test animal is able to recognize an individual (e.g. rodents (reviewed by [84]), goats [48], sheep [68], [69], [85], [86], [87], cattle [70], horses (*Equus caballus*) [71], [72] and pigs (*Sus scrofa*) [88]). While data is generally lacking on the social recognition abilities of goat kids, predictions may be extrapolated from studies on lambs. Porter et al. [87] reported that when 3 week old lambs were first tested with an unfamiliar companion, they became less distressed when subsequently isolated with a lamb they had been housed with for only 5 or 17 days. Studies conducted by Ligout et al. found test lambs vocalized less (a behavior indicative of stress) when paired with familiar lambs than with unfamiliar lambs at 2–3 weeks old [74], even after being separated from the familiar lamb for 5 days [73]. However, it may be that the level of fear was higher in the kids than the motivation to seek comfort in their companions or to distinguish between the stimuli kids. Alternatively, as the stimuli kids were not acclimated to being confined to a test cage, the behavior of the stimuli kids may have affected the test kid by encouraging or discouraging the test kid from interacting with them. While there may have been subtle behaviors made by the stimuli kids, there were no indications that the familiarity of the stimuli kids nor number of vocalizations made by each stimulus kid affected the behavior of the test kid; therefore, we find this unlikely. Additionally, a lack of differentiated responses towards the familiar and unfamiliar stimuli may not necessarily imply a lack of ability to recognize an individual [84]. The visual, olfactory, auditory and limited tactile access between the test kid and stimuli kids may have been sufficient in allowing the test kid to identify the stimuli, be comforted by their presence, then move on to investigate the unfamiliar test arena. In a similar study, Briefer and McElligott [89] demonstrated that both mother goats and kids were able to recognize the vocalizations of each other when the kids were just 1 week of age; subsequently, the mothers retained the ability to remember the vocalizations of their kids up to 13 months after weaning [90]. In similar studies, Nowak [86] found lambs were able to recognize their sibling from an unfamiliar lamb at a distance when tested 1 week after birth and Ligout et al. found visual [85] or olfactory [69] cues were sufficient for 5–6 and 2–3 week old lambs, respectively. However, in line with our results, the responses to familiar (non-sibling) and unfamiliar lambs were similar indicating lambs have a stronger bond with their siblings. Nonetheless, Lickliter [57] found kids were in closer proximity to non-sibling age-mates than siblings during 9 of the first 15 weeks of life which may indicate goat kids have weaker bonds with siblings than lambs. We, therefore, expected the kids to have shown the ability to recognize a non-sibling but familiar kid from an unfamiliar kid at 5 weeks of age.

We found that an increase in density led to an increase in agonistic behaviors for the mothers of the kids used in this study; however, the increase in agonistic behaviors did not negatively affect blood cortisol levels, weight gain or kid production data in the mothers [30]. It is important to note that not only do different stressors have different effects on animals [6] but different species respond to the same stressors in different ways and some may be more resilient than others [8]. Goats, in particular, are adapted to harsh environments due to pressures during natural evolution and domestication [91]. The natural environment of the ancestor of domestic goats is harsh with limited food resources spread over vast rocky terrains [11]. The characteristics of these conditions lead to high competition for resources and preserving rank in dominance that, in turn, leads to frequent and intense agonistic interactions between conspecifics [24], [52], [79]. Even though, in an established group, the social status of an adult female goat remains stable throughout her life [52], [79], agonistic behaviors in goats can be more quite frequent and aggressive than other ungulate species [11], [24], [26], [28]. In addition, the characteristics of the ruminant placenta may aid in protecting the fetus from the influence of maternal hormones in cases of extreme stress [42]. These results coupled with the evolutionary history of goats suggest goats are able to habituate to some extent to living in deficient environmental conditions, such as environments with high animal densities and social stress, at least in terms of reproductive success [30]. The finding that prenatal social stress still had an effect on the fear responses and sociality suggests these may be indicators of the direct fitness of their mothers and long-term abilities of goat kids to survive and reproduce.

## Conclusion

In the current study on the effects of prenatal social stress in goats, we conclude that since the kids from the highest prenatal density of 1.0 m^2^ were more fearful than the kids from lower prenatal densities, this density presented a moderate level of stress. The fact that these effects were more pronounced in females than males is important because it is females that are predominately recruited to the breeding stock of dairy goats. There is also a need to study the longitudinal prenatal effects of high stocking densities as negative effects may be compounded over multiple generations as there is evidence that stress via crowding may affect productivity for at least two generations [19]. In light of the fact that goats are often stocked at densities higher than 1.0 m^2^ in Europe, based on previous findings by Andersen et al. [11], [26], [27], we recommend that goats are kept in larger, stable group sizes and provided with multi-level resting spaces if lower stocking densities are not a possibility for farmers.

## Supporting Information

Video S1 (MPG)

Video S2 (MPG)

Checklist S1 (DOC)
